# Colorimetric Textile Sensor for the Simultaneous Detection of NH_3_ and HCl Gases

**DOI:** 10.3390/polym12112595

**Published:** 2020-11-04

**Authors:** Young Ki Park, Hyun Ju Oh, Jong Hyuk Bae, Jee Young Lim, Hee Dong Lee, Seok Il Hong, Hyun Sik Son, Jong H. Kim, Seung Ju Lim, Woosung Lee

**Affiliations:** 1 Test-Bed Research Center, Korea Dyeing & Finishing Technology Institute (DYETEC), Daegu 41706, Korea; parkyk@dyetec.or.kr (Y.K.P.); hsson95@dyetec.or.kr (H.S.S.); 2 Advanced Textile R&D Department, Korea Institute of Industrial Technology (KITECH), Ansan 15588, Korea; hjoh33@kitech.re.kr (H.J.O.); baejh@kitech.re.kr (J.H.B.); specialg@kitech.re.kr (J.Y.L.); lhd0121@kitech.re.kr (H.D.L.); redstone@kitech.re.kr (S.I.H.); 3 Department of Molecular Science and Technology, Ajou University, Suwon 16499, Korea; 4 Department of Advanced Materials Engineering for Information & Electronics, Kyung Hee University, Yongin 17104, Korea

**Keywords:** textile sensor, gas detection, screen printing, pH sensitivity, durability

## Abstract

For the immediate detection of strong gaseous alkalis and acids, colorimetric textile sensors based on halochromic dyes are highly valuable for monitoring gas leakages. To date, colorimetric textile sensors for dual-gas detection have usually been fabricated by electrospinning methods. Although nanofibrous sensors have excellent pH sensitivity, they are difficult to use commercially because of their low durability, low productivity, and high production costs. In this study, we introduce novel textile sensors with high pH sensitivity and durability via a facile and low-cost screen-printing method. To fabricate these textiles sensors, Dye 3 and RhYK dyes were both incorporated into a polyester fabric. The fabricated sensors exhibited high detection rates (<10 s) and distinctive color changes under alkaline or acidic conditions, even at low gas concentrations. Furthermore, the fabricated sensors showed an outstanding durability and reversibility after washing and drying and were confirmed to contain limited amounts of hazardous materials. Thus, our results show that the fabricated textile sensors could be used in safety apparel that changes its color in the presence of harmful gases.

## 1. Introduction

Ammonia (NH_3_) and hydrochloric acid (HCl) are used in various industries, such as for industrial cleaning, food and chemical manufacturing, as a refrigerant (NH_3_), and for pickling (HCl) [1,2,3]. Despite their usefulness, NH_3_ and HCl are toxic substances, and inhalation or contact with the skin or eyes can cause irritation and burns [4,5,6,7,8]. According to the Occupational Safety and Health Administration (OSHA), the permissible exposure limits (calculated as 8 h time-weighted averages) of NH_3_ and HCl are 50 and 5 ppm, respectively [9]. However, gaseous NH_3_ and HCl are difficult to detect with the naked eye because they are colorless and spread quickly. Therefore, continuous monitoring is necessary to detect gas leaks in industries that store, handle, or manufacture these toxic substances.

To detect gas leaks, electrochemical, catalytic combustion, semiconductor, and infrared gas sensors are usually used in various industries. Currently, as the demand for portable gas sensors has been increasing, the miniaturization of these gas sensors is in progress, but their inconvenient portability, difficulties in reducing their power consumption, and high manufacturing costs are persisting obstacles. To overcome these problems, sensors directly applied to textiles are widely studied, while most of these studies are based on electro resistive sensors [10,11,12,13,14,15]. However, such sensors require a power source, which makes them difficult to use in safety apparel.

Recently, colorimetric textile sensors based on halochromic dyes have raised attention because naked-eye-detectable color changes can immediately alert workers of chemical leakages without power sources being involved [16,17,18,19,20,21]. However, the relatively low pH sensitivity and stability of these sensors reduces the detection performance at a low gas concentration, limiting their wide application. To improve the low pH-sensitivity of textile sensors, most of these sensors [22,23,24,25] have been fabricated with the electrospinning method because of their large specific surface area advantageous for pH sensitivity. For example, Agarwal et al. [24] fabricated universal pH sensing nanofibrous sensors using various halochromic dyes with Nylon 6. Pakolpakçıl et al. [26] used natural halochromic dye with a sodium alginate and polyvinyl alcohol mixture to fabricate pH-indicating nanofibrous sensors. Getmeyer et al. [27] fabricated nanofibrous sensors using a combination of electrospinning and sol-gel methods to detect gaseous NH_3_ and HCl. Moreover, Jeevarathinam et al. [28] and Suleymanov et al. [29] reported detection via an aggregation-induced emission of the colorant. Previous studies mainly focused on the detection performance of the fabricated sensors with respect to liquid or gaseous alkalis and acids. However, for the commercial use of textile sensors, pH sensitivity as well as reusability, washability, productivity, and production costs should be considered.

As an alternative to this approach, conventional dyeing and printing methods have the advantages of simplicity, low production costs, and good durability. In the dyeing method, dyes are generally applied to the fiber surface and interior. In contrast, in the printing method, dyes are mostly applied to the surface of the textile, resulting in a higher reaction rate and higher reactivity. Thus, textile sensors fabricated by the printing method typically exhibit better pH-sensitivity than those fabricated by the dyeing method.

The aim of this study was to fabricate colorimetric textile gas sensors that can detect both gaseous strong alkalis and acids with superior reversibility, reusability, and washability. To the best of our knowledge, printing-based, durable, high-sensitive colorimetric textile gas sensors have not been reported yet. In this study, we fabricated the textile sensors using a conventional printing method for dual-gas detection based on mixtures of Dye 3 [30] and RhYK [31], which are hydrophobic halochromic dyes with superior pH sensitivity and durability. The dual-gas detection performance of the fabricated textile sensors was investigated at various gas concentrations using a homemade gas tester connected with a computer color matching system. In addition, durability, reversibility, and hazardous material tests on the fabricated textile sensors were conducted.

## 2. Materials and Methods

### 2.1. Materials

Polyester adjacent fabric (ISO 105-F04) was obtained from Testfabrics Korea, Inc. (Ansan, Korea). 2-(3,5,5-trimethylcyclohex-2-enylidene)malononitrile, 2-hydroxy-4-methoxybenzaldehyde, 2-hydroxycarbazole, phthalic anhydride, zinc chloride, ethyl cellulose, ethanol, piperidine, dimethylformamide, acetonitrile, sodium chloride, ammonium hydroxide, hydrochloric acid, formic acid, triethyl citrate, formaldehyde, ethyl ether, methylene chloride, methanol, acetone, and toluene were purchased from Thermo Fisher Scientific Korea (Seoul, Korea) and used without further purification.

### 2.2. Dye Synthesis

#### 2.2.1. Synthesis of 2-{3-[2-(2-hydroxy-4-methoxy-phenyl)-vinyl]-5,5-dimethyl-cyclohex-2-enylidene}-malononitrile (Dye 3)

Dye 3, for alkaline gas detection, was synthesized as described in our previous report [30]. After dissolving, equimolar amounts of 2-(3,5,5-trimethylcyclohex-2-enylidene)malononitrile and 2-hydroxy-4-methoxybenzaldehyde were dissolved in ethanol, the solution was titrated with piperidine. The reaction mixture was refluxed at room temperature for 7 h and then the product was isolated by filtration and purified (60% yield).

EA: anal. calcd for C_19_H_20_N_2_O_2_: C, 74.98; H, 6.29; N, 8.74; found: C, 74.50; H, 6.41; N, 8.55. ^1^H NMR (500 MHz, DMSO-d_6_): δ 1.01 (s, 6H), 2.58 (s, 2H), 3.32 (s, 2H), 3.74 (s, 3H, –OCH_3_), 6.45–6.48 (m, 2H), 6.75 (s, 1H), 7.20 (d, *J* = 16.2 MHz, 1H), 7.41 (s, *J* = 16.25 MHz, 1H), 7.64 (d, *J* = 8.6 MHz, 1H), 10.25 (s, 1H, –OH). MS: [M]^+^ = 320. mp: 194–195 °C.

#### 2.2.2. Synthesis of 5′,9′-dihydro-3H-spiro[isobenzofuran-1,15′-pyrano [2,3-b:6,5-b’]dicarbazol]-3-one (RhYK)

RhYK, for acidic gas detection, was synthesized as described in our previous report [31]. 2-hydroxycarbazole (0.01 mol), zinc chloride (0.01 mol), and phthalic anhydride (0.015 mol) were heated at 160 °C for 24 h under solventless conditions. After cooling to room temperature, DMF (20 mL) was added and the reaction mixture was stirred for 30 min. The mixture was then added dropwise to water (200 mL) containing 10 wt% NaCl and 3 wt% HCl while stirring. The resulting purple precipitate was isolated by filtration and purified by silica gel column chromatography with an acetonitrile/water (containing 0.1% formic acid) gradient system (99:1–50:50) (74.8% yield).

EA: anal. calcd for C_32_H_19_N_2_O_3_^+^: C, 74.64; H, 3.90; N, 5.44; O, 9.32; found: C, 74.17; H, 3.93; N, 5.31; O, 11.54. ^1^H NMR (500 MHz, DMF-*d*_7_, TMS): δ 7.110 (t, 2H), 7.377 (t, 2H), 7.510 (d, 1H), 7.543 (d, 2H), 7.562 (s, 1H), 7.805 (s, 2H), 7.868 (t, 1H), 7.896 (t, 1H), 8.067 (d, 2H), 8.173 (d, 1H), 11.725 (s, 1H). MS: [M]^+^ = 479.139. FT-IR (cm^−1^): 1718 (C=O), 1615–1457 (C–H), 3400–3200 (O–H). Decomposition temperature: 220 °C.

### 2.3. Fabrication of Colorimetric Textile Sensors

**Scheme 1:**
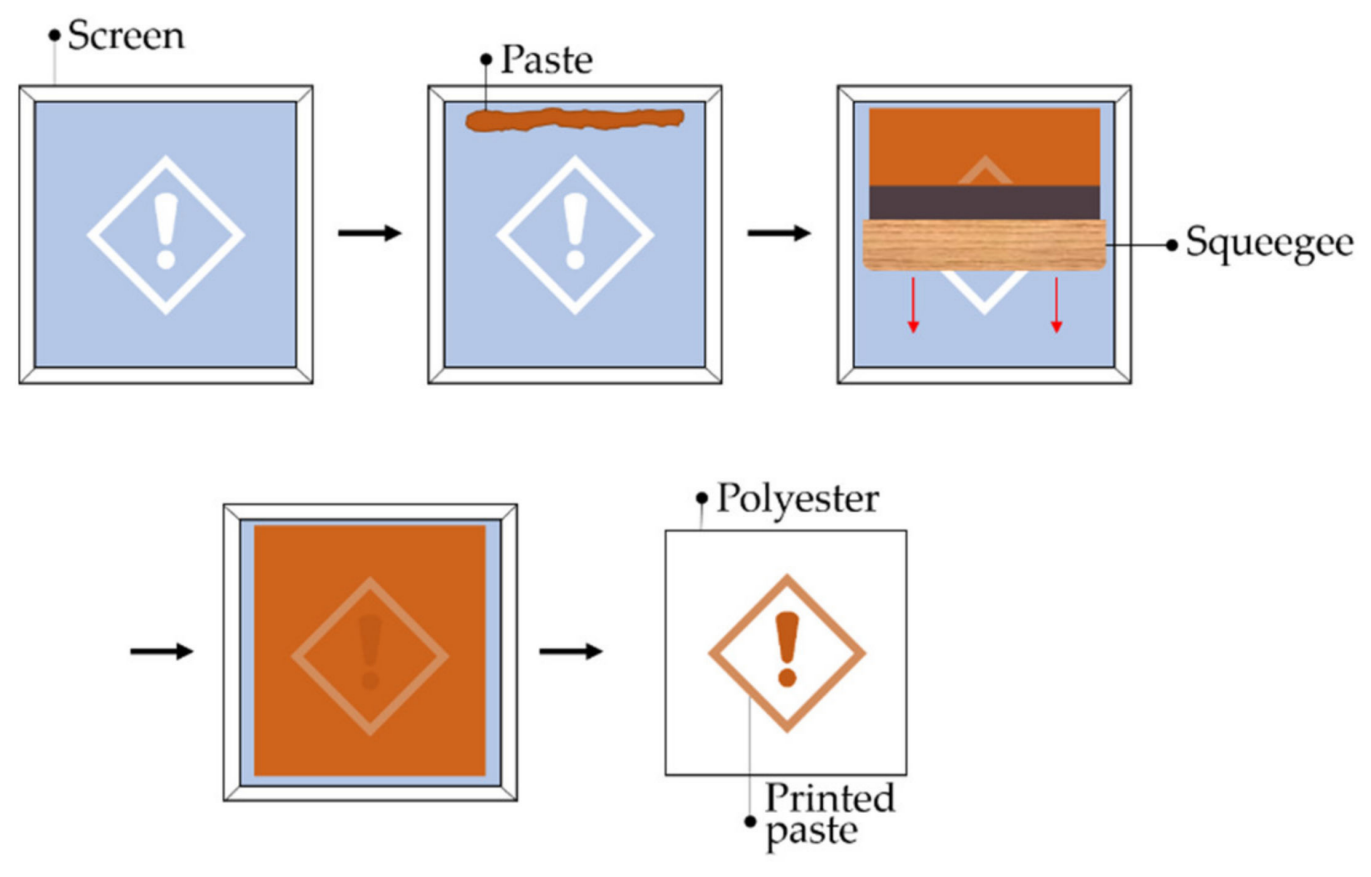
Schematic diagram of the screen-printing method.

Ethyl cellulose (2 g) was dissolved in ethanol (20 g). After complete dissolution, a single dye (0.1 g of Dye 3 or RhYK) or mixed dyes (0.1 g of Dye 3 and 0.06 g of RhYK) and a plasticizer (triethyl citrate; 3 g) were added. Then, textile sensors were fabricated by screen printing using the polyester fabric as a substrate and a screen with a mesh size of 100 µm (Scheme 1). After printing, the sensors were cured at 80 °C for 5 min.

### 2.4. Characterization

The surface morphology of the fabricated textile sensors based on Dye3/RhYK was observed by field emission scanning electron microscopy (FE-SEM) (SU8010, Hitachi Co, Tokyo, Japan) with an acceleration voltage of 10 kV after sputter coating with platinum (Pt).

### 2.5. Gas Detection Performance

**Figure 1:**
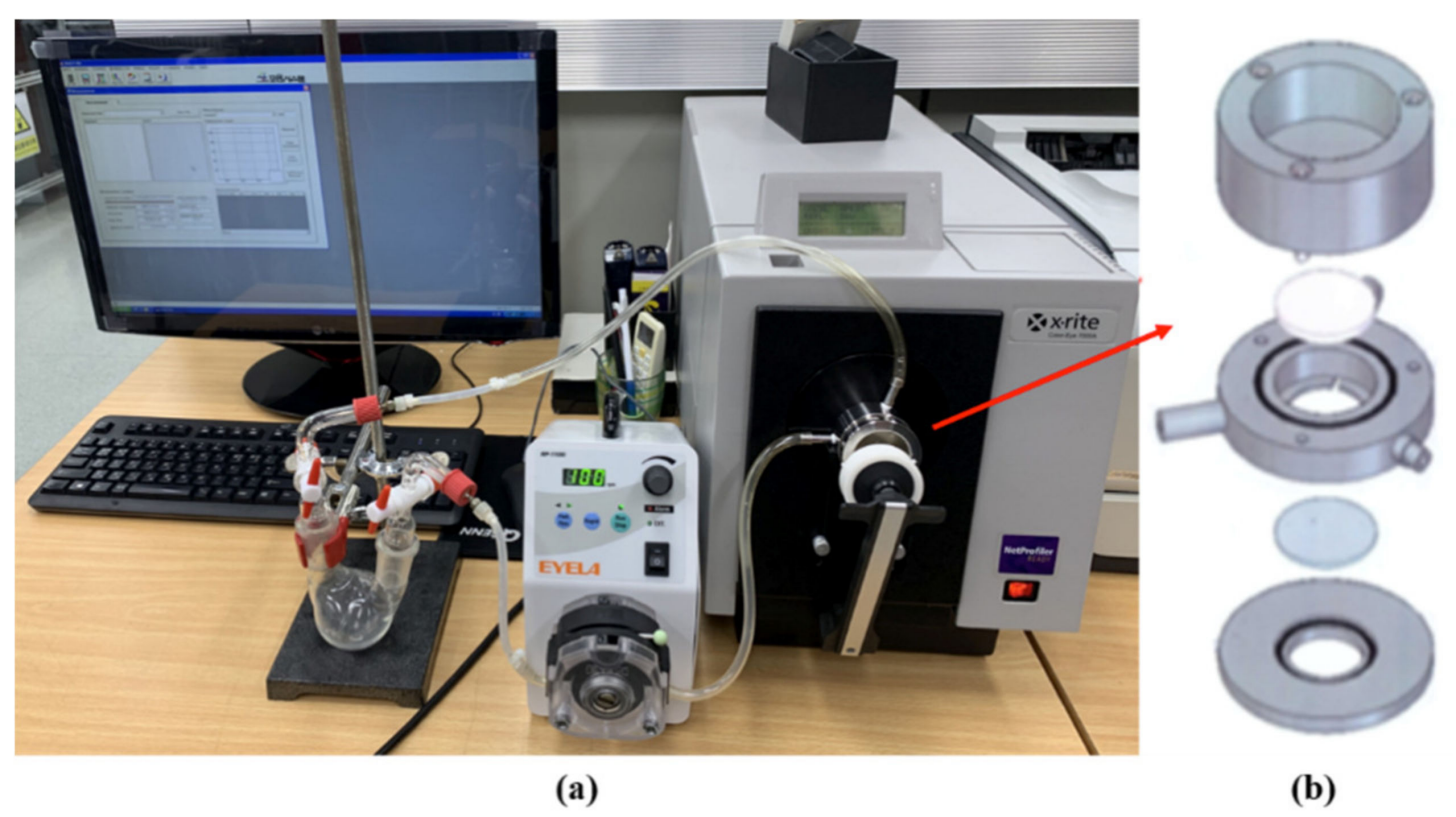
Color measurement system connected to a gas flow device for dynamic color change measurements: (**a**) Optical image of the entire system; (**b**) Schematic diagram of the chamber with a loaded sample.

The detection performance of the fabricated textile sensors was characterized using a gas test system, as described in our previous report [32]. The gas test system (Figure 1) allowed the color change of the textile sensors when exposed to gas to be measured in real time. The sensors were placed in a stainless-steel chamber and the gas was circulated through the Teflon tube connected to the chamber. Because the internal volume of the entire system was known, the concentration of the gas could be controlled by the amount of liquid injected into the two-neck round-bottom flask. The liquid in the flask was evaporated using a heat gun, and the gas was circulated through the entire system using a peristaltic pump. After exposure to the gas, the dynamic color change of the textile sensors was measured using a ColorEye 7000A spectrophotometer (X-rite, Grand Rapids, MI, USA).

To measure the color strength of the fabricated textile sensors, K/S values were calculated. According to the Kubelka–Munk theory, the K/S value can be obtained from the surface reflectance at the maximum absorption wavelength, as given by Equation (1) [33]:

(1) $$K/S\;=\;{\left(1-R\right)}^{2}/2R,$$

where K is the absorption coefficient, S is the scattering coefficient, and R is the reflectance (0 < R ≤ 1). The K/S values of the textile sensors were determined using a spectrometer with a D65 standard illuminant and a 10° standard observer.

Using NH_3_ or HCl gas in the concentration range of 1 to 100 ppm, the color change of the textile sensors was measured every 10 s for 2 min. The color difference value (ΔE) represents the relative difference between two colors and was calculated using Equation (2) [34]:

(2) $$\Delta E\;=\;\sqrt{{\left(\Delta L^{*}\right)}^{2}+{\left(\Delta a^{*}\right)}^{2}+{\left(\Delta b^{*}\right)}^{2}},$$

where L* is the lightness, a* is the red/green value, and b* is the yellow/blue value.

### 2.6. Volatile Organic Compound (VOC) Test

Each VOC (formaldehyde, ethyl ether, methylene chloride, methanol, ethanol, acetone, or toluene; 10 mL) was placed in a vial. After heating the VOC to above its boiling point (1–5 °C) for 3 min, the textile sensor was placed at the mouth of the vial to observe the color change when exposed to VOC gases.

### 2.7. Fastness Test

The fabricated textile sensors were subjected to a wash fastness test according to method A of ISO 105C10 [35]. The textile sensors were washed for 30 min at 40 °C. For this test, 5 g/L of SDCE standard soap (SDCE Type 1, SDC Enterprises Ltd., Holmfirth, UK) was used, and the bath ratio was 50:1. The sample dimensions were 100 × 40 mm. The shade change and the staining of adjacent fabrics were rated using the appropriate ISO gray scale.

### 2.8. Hazardous Materials Test

The presence of hazardous materials in the fabricated textile sensors was investigated using Korean Industrial Standards. The arylamine content was analyzed using gas chromatography–mass spectrometry (GC-MS) according to KS K 0147 [36]. The content of allergenic disperse dyes was measured using high-performance liquid chromatography (HPLC) according to KS K 0736 [37]. The formaldehyde content was measured using ultraviolet–visible (UV-Vis) spectroscopy according to KS K ISO 14184-1 [38]. The pH of the fabricated sensors was measured with a pH meter according to KS K ISO 3071 [39]. The total Pb and Cd contents were tested according to Ministry of Trade, Industry and Energy (MOTIE) notification No. 2019-201 “Common safety standards for children’s products”.

### 2.9. Reversibility Test

To evaluate the reusability, the fabricated sensors were subjected to a gas test (10 ppm NH_3_ or HCl), washed with water (2 min), and then dried (50 °C, 5 min). This process was repeated five times.

## 3. Results and Discussion

### 3.1. Gas Detection by Textile Sensors Based on Single Dyes

Before fabricating the textile sensor based on mixed dyes for dual-gas detection, the detection performance of single-dye-based textile sensors was investigated. The textile sensors based on a single dye were fabricated via the screen-printing method using a printing paste prepared by mixing a binder, dye, solvent, and plasticizer. To use it for garment, binder, solvent, and plasticizer were selected in consideration of biocompatibility as well as compatibility with dyes.

#### 3.1.1. Gas Detection Performance of the Textile Sensor Based on Dye 3

**Figure 2:**
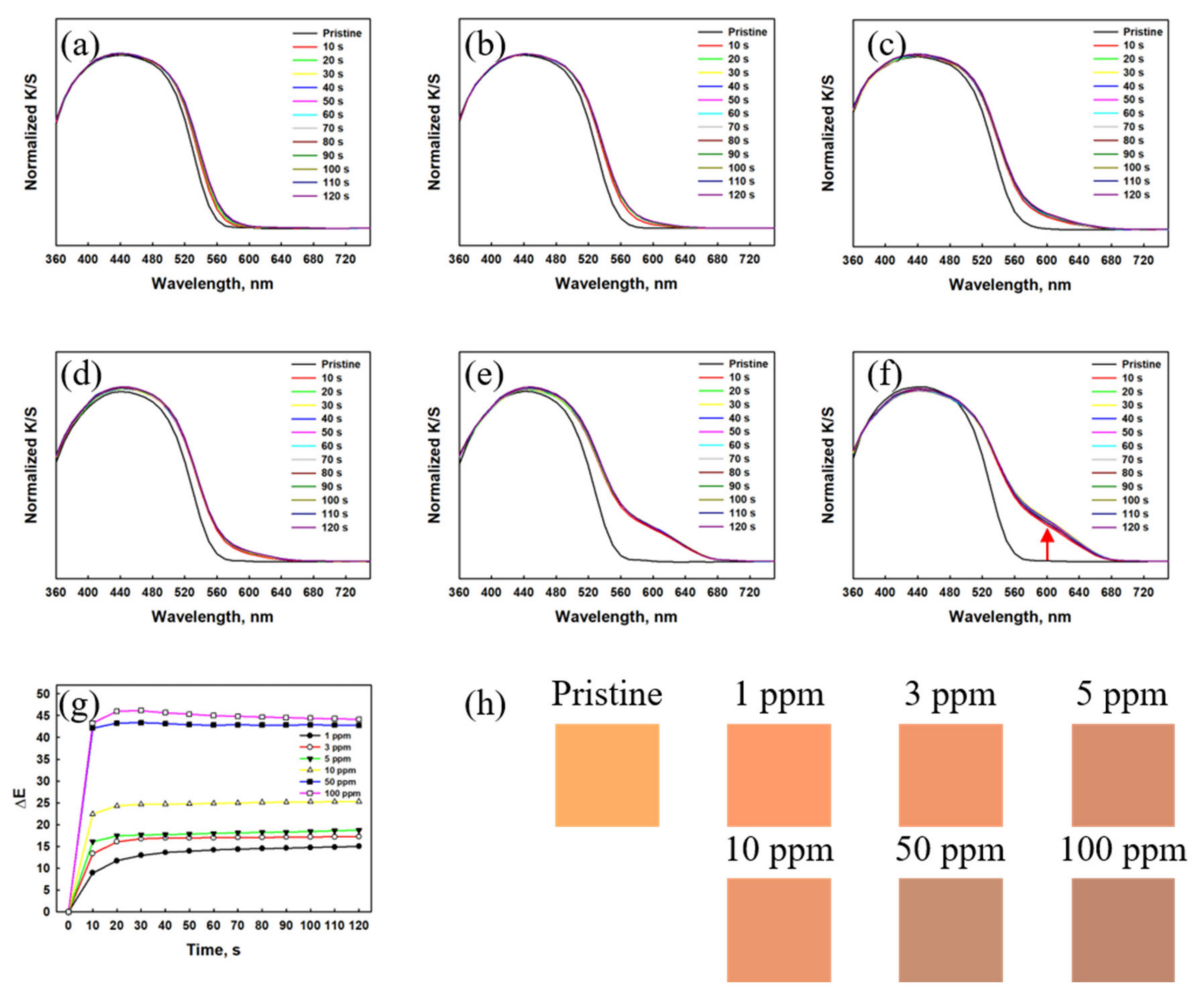
Color data for the textile sensor based on Dye 3 after exposure to various concentrations of gaseous NH_3_: (**a**–**f**) Time-dependent K/S values upon exposure to 1, 3, 5, 10, 50, and 100 ppm NH_3_; (**g**) Time-dependent color differences; (**h**) Color changes.

**Scheme 2:**
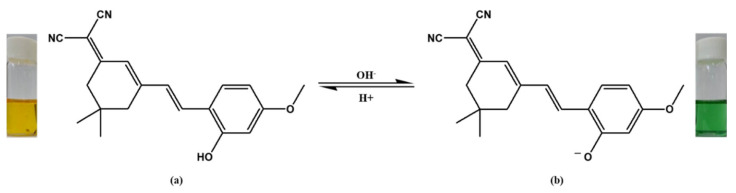
Halochromism of Dye 3 in solution: (**a**) Dye 3 (yellow); (**b**) Dye 3a (green).

Figure 2 shows the color data for the textile sensor based on Dye 3 after exposure to gaseous NH_3_ at various concentrations. In general, when the color difference value (ΔE) is 5 or higher, observers can recognize two distinct colors [30]. Therefore, the detection rate was determined as the time required for the color difference value to reach 5, and the reactivity was evaluated using the color difference value when the color change reached saturation. As shown in Figure 2a–f,h, when the fabricated sensor was exposed to NH_3_ gas, the color changed from yellow to brown and the K/S value at ~600 nm gradually increased. As shown in Scheme 2, under alkaline solvent conditions, the hydroxyl group of Dye 3 is deprotonated, resulting in a bathochromic shift (yellow to green). However, in the textile sensor, the sensor is yellow under neutral conditions and brown under alkaline conditions. This color difference between the solvent state and the printed state can be explained as follows. First, it is well known that the microenvironment affects dye–fiber interactions and thus the halochromic properties of dyes [40,41,42]. Second, as not all dye molecules react with NH_3_, a mixed color originating from reacted and unreacted dye molecules is observed upon exposure to NH_3_ gas. Third, when comparing the Absorbance-Wavelength graph in the textile and solution state, it can be seen that the peak in the textile appears broader under neutral and alkaline conditions. This is because the dyes in the textile are more aggregated than in the solution state, so that more electronic transition occurs due to more interactions between the dyes. As can be seen in Figure 2a–f of the textile state, this broad peak appears in the overall visible light range under alkaline conditions, so that the color of the sensor becomes brown. As shown in Figure 2g, the detection rate of the Dye 3 sensor was very fast (within 10 s), and the saturated color difference value (ΔE_max_) was greater than 10, indicating excellent detection performance. The initial increase of the ΔE value occurred more rapidly as the gas concentration increased, indicating that the detection rate increased at higher gas concentrations. This behavior is considered to originate from improved contact between the gas and the dye as the gas concentration increases. These results confirm that the conventional printing method can be applied to manufacture a textile sensor for detecting NH_3_ gas.

#### 3.1.2. Gas Detection Performance of the Textile Sensor Based on RhYK

**Figure 3:**
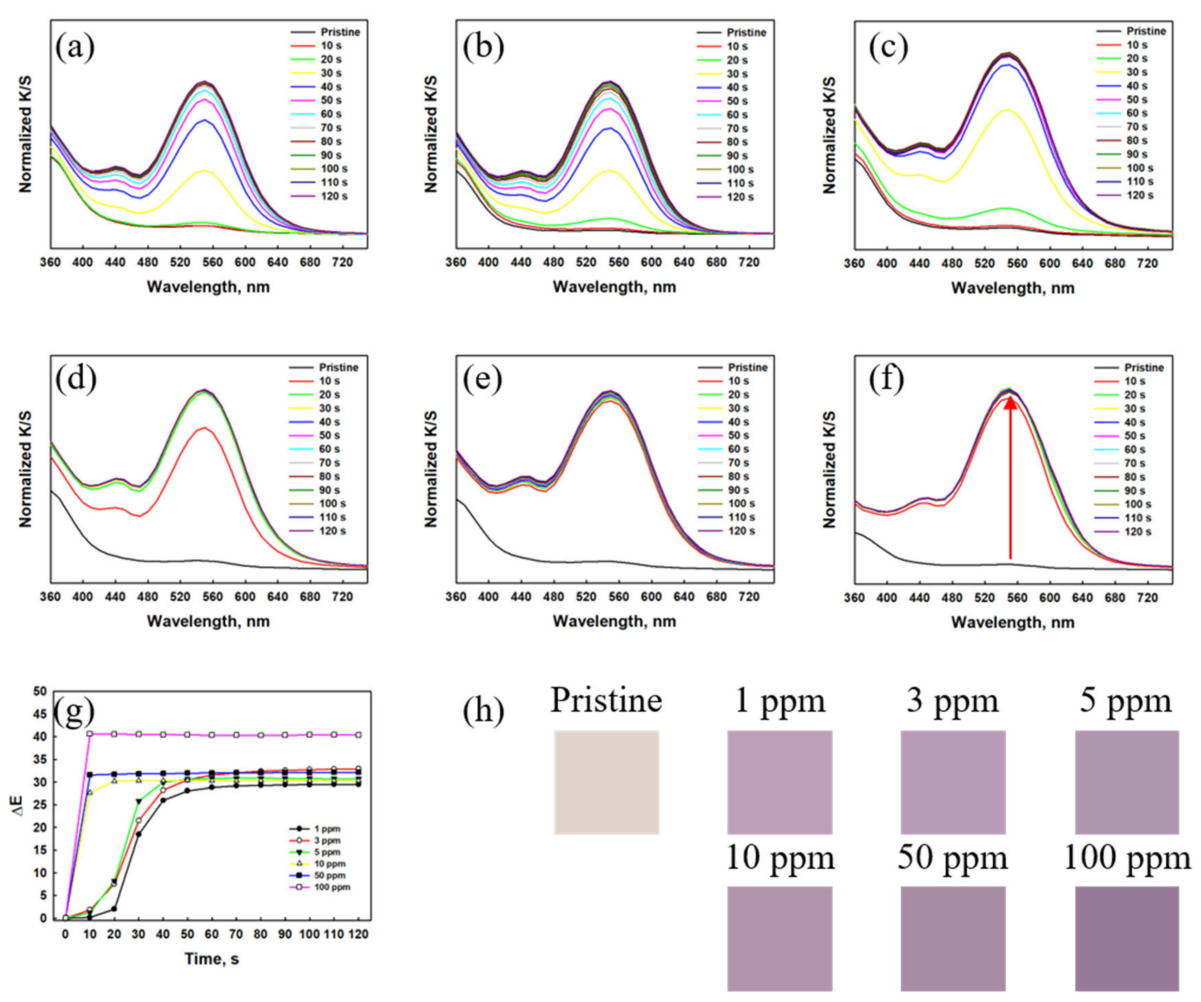
Color data for the textile sensor based on RhYK after exposure to various concentrations of gaseous HCl: (**a**–**f**) Time-dependent K/S values upon exposure to 1, 3, 5, 10, 50, and 100 ppm HCl; (**g**) Time-dependent color differences; (**h**) Color changes.

**Scheme 3:**
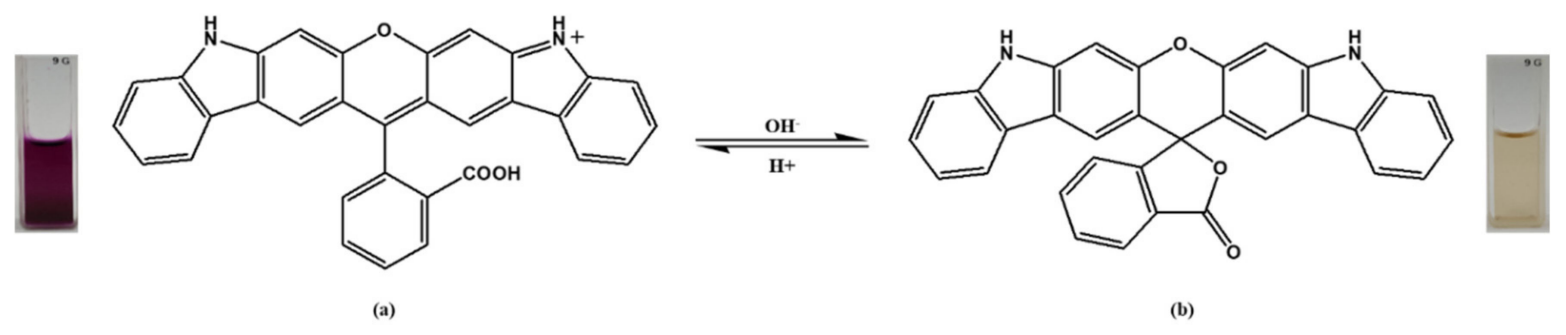
Halochromism of RhYK in solution: (**a**) Ring-opened form (violet-red); (**b**) Spirolactone form (colorless).

Figure 3 shows the color data for the textile sensor based on RhYK after exposure to gaseous HCl at various concentrations. As shown in Figure 3a–f,h, upon exposure to HCl gas, the sensor changed from colorless to purple as the K/S value at ~550 nm gradually increased. This color change can be explained by the halochromic properties of the dye, as shown in Scheme 3. Under acidic conditions, RhYK undergoes a ring-opening reaction, resulting in a bathochromic shift (colorless to violet-red). As shown in Figure 3g, the detection rate of the RhYK sensor was very fast (within 10 s), and the ΔE_max_ value was greater than 20, indicating excellent detection performance. Similar to Dye 3, the initial increase of the ΔE value occurred more rapidly as the gas concentration increased, indicating that the detection rate increased at higher gas concentrations. These results confirm that the conventional printing method can be applied to manufacture a textile sensor for detecting HCl gas.

### 3.2. Dual-Gas Detection by Textile Sensors Based on Mixed Dyes (Dye 3/RhYK)

**Figure 4:**
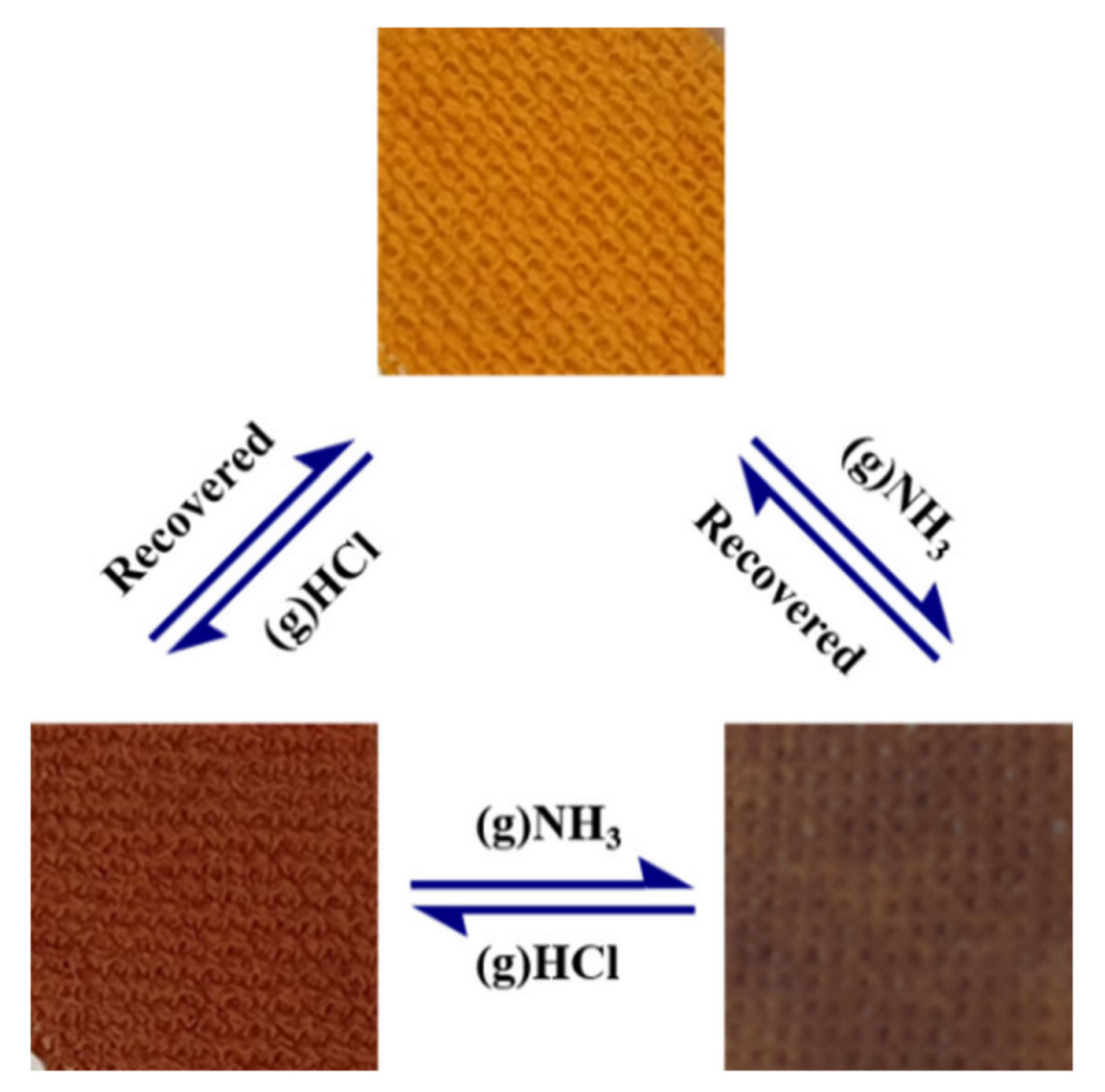
Color of the textile sensor based on mixed dyes before and after exposure to gaseous HCl and NH_3_.

**Figure 5:**
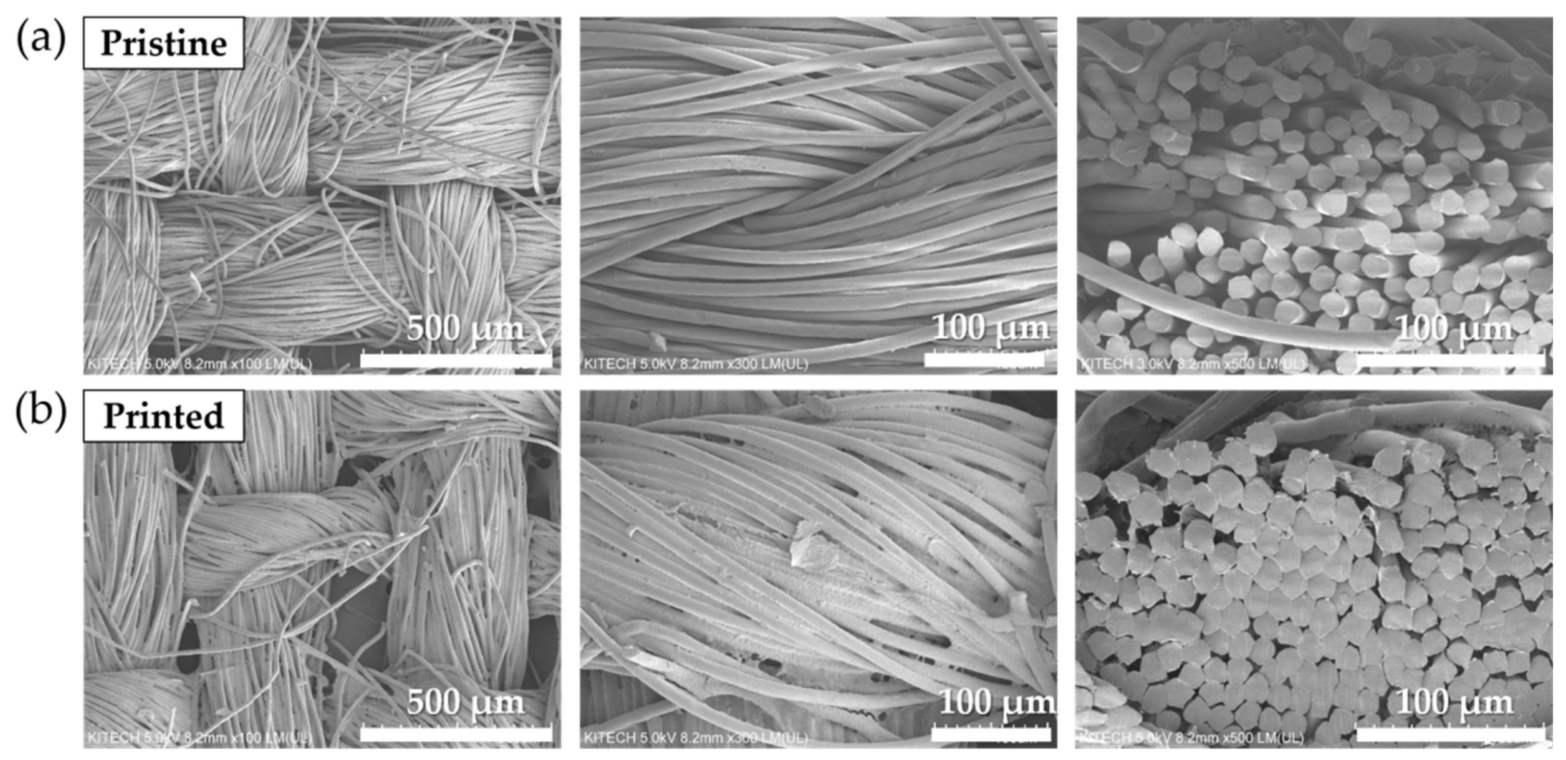
FE-SEM image of the fabricated textile sensor with mixed-dyes (Dye 3/RhYK): (**a**) Pristine polyester fabric, (**b**) Printed polyester fabric.

To investigate the possibility of dual-gas detection, a gas test was conducted for a textile sensor fabricated using a mixture of Dye 3 and RhYK (Figure 4). To fabricate the mixed-dye sensor, the amount of RhYK was decreased from 0.1 g to 0.06 g because of the low solubility this dye. The surface morphologies of the fabricated textile sensors are shown in Figure 5. A thinly formed binder on the surface of the fiber confirmed that the mixed dyes were successfully printed.

#### 3.2.1. NH_3_ Gas Detection by the Mixed-Dye Sensor

**Figure 6:**
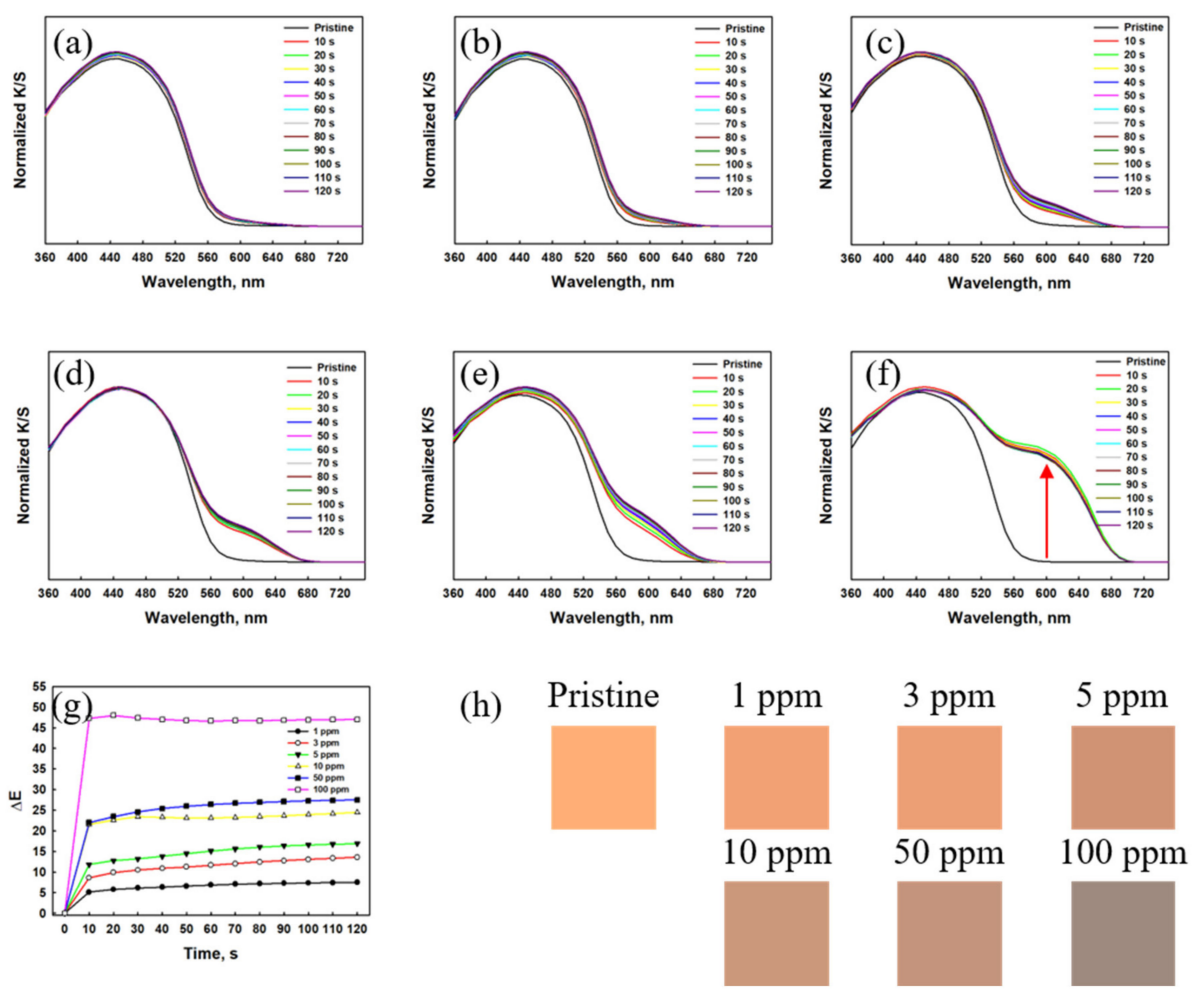
Color data for the textile sensor based on mixed dyes (Dye 3/RhYK) after exposure to various concentrations of gaseous NH_3_: (**a**–**f**) Time-dependent K/S values upon exposure to 1, 3, 5, 10, 50, and 100 ppm NH_3_; (**g**) Time-dependent color differences; (**h**) Color changes.

Figure 6 shows the color data for the textile sensor based on mixed dyes (Dye 3/RhYK) after exposure to gaseous NH_3_ at various concentrations. As shown in Figure 6a–f,h, the color of the mixed-dye sensor was similar to that of the single dye (Dye 3) sensor under neutral and alkaline conditions. This is because RhYK is colorless under these conditions. As shown in Figure 6g, the reactivity of the mixed-dye sensor was lower than that of the Dye 3 sensor at the same NH_3_ concentrations. Under neutral conditions, the ring-opened and spirolactone forms of RhYK coexist (the spirolactone form is predominant). As this small amount of ring-opened RhYK also reacts with NH_3_ gas, the reaction of Dye 3 with NH_3_ gas in the mixed-dye sensor is relatively reduced compared to that in the Dye 3 sensor. However, despite this decrease in detection performance, the detection rate of the sensor remained very fast (within 10 s), and the ΔE_max_ value was 5 or higher at all gas concentrations of NH_3_. These results confirm that NH_3_ gas can be detected visually using the mixed-dye textile sensor.

#### 3.2.2. HCl Gas Detection by the Mixed-Dye Textile Sensor

**Figure 7:**
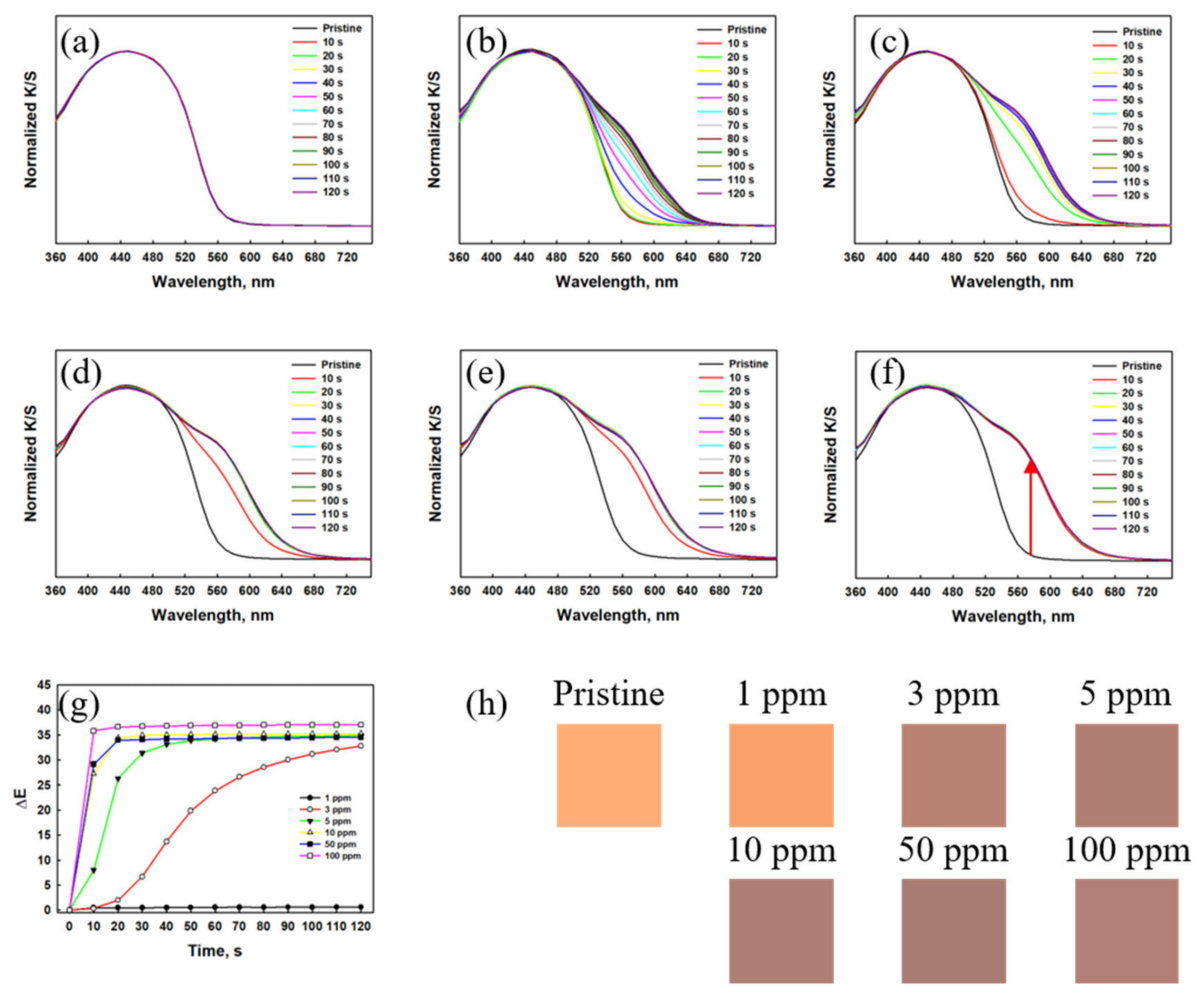
Color data for the textile sensor based on mixed dyes (Dye 3/RhYK) after exposure to various concentrations of gaseous HCl: (**a**–**f**) Time-dependent K/S values upon exposure to 1, 3, 5, 10, 50, and 100 ppm HCl; (**g**) Time-dependent color differences; (**h**) Color changes.

Figure 7 shows the color data for the textile sensor based on mixed dyes after exposure to gaseous HCl at various concentrations. As shown in Figure 7a–f,h, the mixed-dye sensor appears yellow and brown under neutral and acidic conditions, respectively. Under acidic conditions, RhYK and Dye 3 are purple and yellow, respectively, which mix to give the reddish-brown color of the mixed-dye sensor. As shown in Figure 7g, the mixed-dye sensor has relatively poor detection performance compared to the single dye (RhYK) sensor, especially at low concentrations. This decreased performance can be explained as follows. First, the amount of RhYK used to fabricate the mixed-dye sensor was reduced from 0.1 g to 0.06 g owing to the low solubility of RhYK in EtOH. Second, similar to RhYK, Dye 3 can react not only NH_3_ but also HCl. Hence, the detection limit of the mixed-dye sensor is higher than that of the single-dye sensor. Despite this decrease in detection performance, the detection rate remained very fast (within 10 s), and the ΔE_max_ value was 30 or higher at all concentrations of HCl, except 1 ppm. This result indicates that HCl gas can be detected visually with the mixed-dye sensor.

### 3.3. Effect of VOCs on Textile Sensors

As shown in Table S1, the effect of various VOCs on the mixed-dye sensor was investigated. As Dye 3 and RhYK were designed to detect alkalis and acids, respectively, they did not show perceptible color changes when exposed to VOC gases. These results indicate that the fabricated textile sensor has high selectivity in gas detection for NH_3_ and HCl.

### 3.4. Wash Fastness of Textile Sensors

The wash fastness of the fabricated sensors using Dye 3 and RhYK dyes was evaluated to confirm the durability of the sensors (Table 1). To investigate the durability of each dye, this test was performed using the single-dye sensors rather than the mixed-dye sensor. As shown in Table 1, both textile sensors exhibit excellent wash fastness, with ratings of 4–5. This good performance might be due to the low depth of shade resulting from the low K/S values on polyester fabric and the hydrophobic nature of the dyes. These results indicate that both textile sensors can be reused after washing and drying without a severe reduction in detection performance caused by dye dropout.

### 3.5. Hazardous Materials in Textile Sensors

Table 2 and Table S2 show the contents of hazardous materials in the textile sensor fabricated with mixed dyes. The content of each of the investigated hazardous materials was below the corresponding detection limit, and the sensor had a near-neutral pH of 7.2. These results indicate that the fabricated sensors are not harmful to the human body and thus can be applied to garments.

### 3.6. Reversibility of Textile Sensors

To evaluate the reusability of the mixed-dye sensor, their reversibility was measured over five NH_3_/HCl gas detection–washing–drying cycles.

#### 3.6.1. Reversibility with NH_3_ Gas

**Figure 8:**
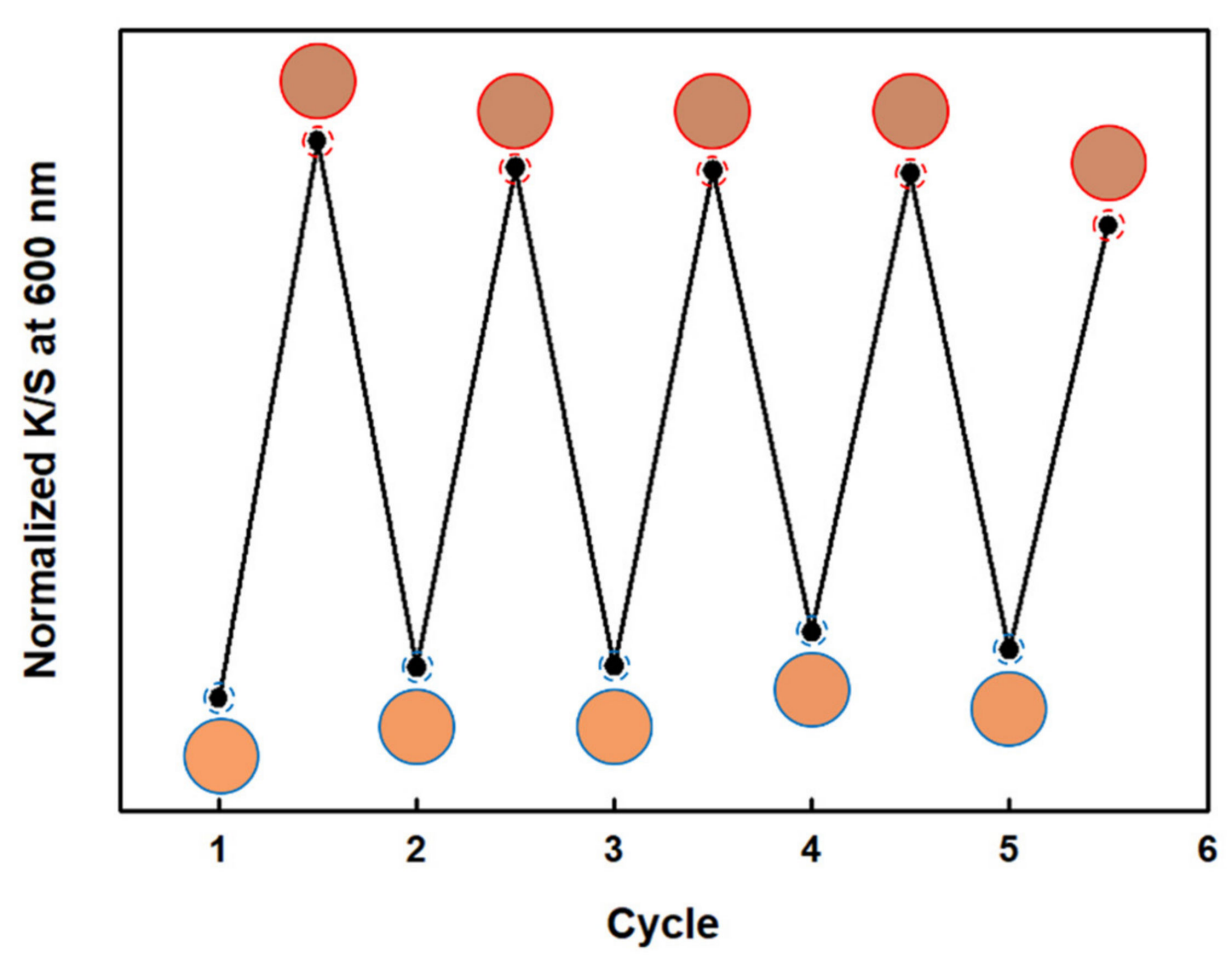
Reversibility of the fabricated textile sensor with NH_3_ gas: (**blue circle**) before exposure; (**red circle**) after exposure.

As the number of NH_3_ gas exposure cycles increased, the final normalized K/S value decreased, while the initial normalized K/S value increased (Figure 8). The increase in the initial normalized K/S value indicates that the sensor did not return to its original color after washing and drying. This behavior could indicate that the washing process was insufficient to convert all the alkali form of the dye into the neutral form. The decrease in the final normalized K/S value indicates that a small amount of dye was removed from the textile sensor during the washing process. However, because the changes in the initial and final normalized K/S values are small, a noticeable color change still occurs, and the detection performance of the dye is considered to be maintained.

#### 3.6.2. Reversibility with HCl Gas

**Figure 9:**
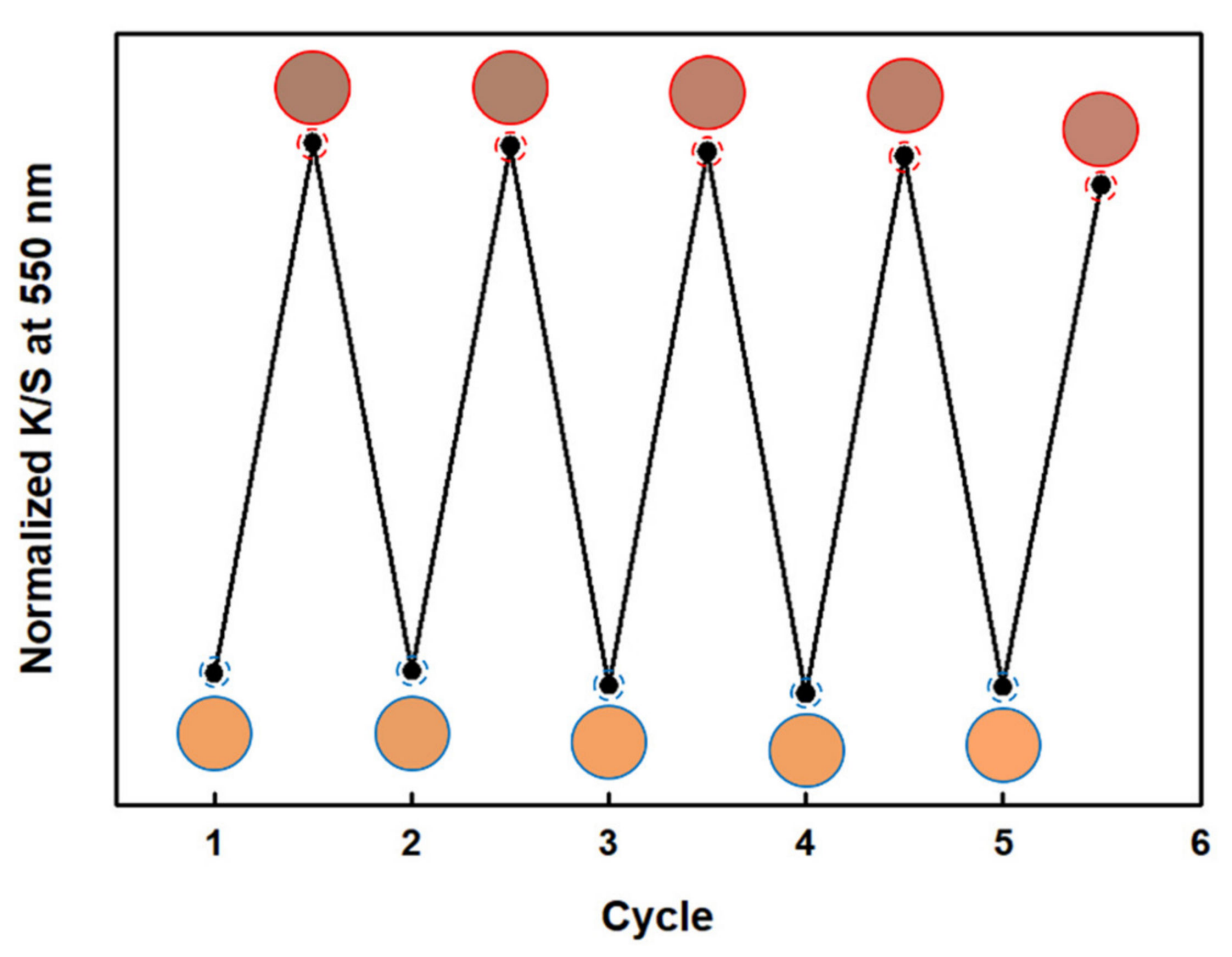
Reversibility of the fabricated textile sensor with HCl gas: (**blue circle**) before exposure; (**red circle**) after exposure.

As shown in Figure 9, the initial and final normalized K/S values remained relatively constant as the number of HCl gas exposure cycles increased. As the changes in the initial and final normalized K/S values are small, even after washing and drying five times, fabricated textile sensor exhibits good reusability for HCl sensing, similar to that observed for NH_3_ sensing.

## 4. Conclusions

In this study, Dye 3 and RhYK, which show high pH sensitivity and durability, were applied to fabricate textile sensors using a screen-printing method. The detection performance of the fabricated sensors was evaluated using gaseous NH_3_ and HCl. The textile sensors based on single dyes exhibited high detection rates (<10 s) and high reactivities, even at low gas concentrations. In the mixed-dye sensor for dual-gas detection, the reactions of the dyes with gaseous NH_3_ and HCl remained rapid, causing distinct color changes. Thus, despite some deterioration in detection performance compared with the single-dye textile sensors, the mixed-dye sensor exhibited an excellent detection rate and reactivity to NH_3_/HCl gas, even at 5 ppm. In addition, owing to the hydrophobic nature of the dyes, the textile sensors showed superior wash fastness, and the good reversibility was observed, even after five washing and drying cycles. Furthermore, the fabricated sensors were confirmed to contain limited amounts of hazardous materials. Consequently, this method of fabricating textile sensors is promising for the production of color-changing garments that are sensitive to harmful gases. For the development of colorimetric textile sensors with high visibility and sensitivity, further studies on colorants changing from colorless to deep colors upon pH variation are needed.

## Figures and Tables

**Table 1:** Wash fastness of fabricated textile sensors.

Dye	Change	Staining					
		A	C	N	P	Ac	W
Dye 3	4–5	4–5	4–5	4–5	4–5	4–5	4–5
RhYK	4–5	4–5	4–5	4–5	4–5	4–5	4–5

A: acetate, C: cotton, N: nylon, P: polyester, Ac: acrylic, W: wool.

**Table 2:** Hazardous material contents of fabricated textile sensor based on mixed dyes.

Arylamines (mg/kg)	Allergenic Disperse Dyes (mg/kg)	Formaldehyde (mg/kg)	pH	Total Pb (mg/kg)	Total Cd (mg/kg)
<5	<20	Not detected	7.2	<10	<10

## Supplementary Materials

The following are available online at https://www.mdpi.com/2073-4360/12/11/2595/s1, Table S1: Effect of VOCs on the fabricated textile sensor based on mixed dyes, Table S2: Content of hazardous materials of fabricated textile sensor.

## References

[1] Chebabe D., Chikh Z.A., Hajjaji N., Srhiri A., Zucchi F. (2003). Corrosion inhibition of Armco iron in 1 M HCl solution by alkyltriazoles. Corros. Sci..

[2] Bhawsar J., Jain P.K., Jain P. (2015). Experimental and computational studies of *Nicotiana tabacum* leaves extract as green corrosion inhibitor for mild steel in acidic medium. Alex. Eng. J..

[3] Shahabuddin M., Sharma A., Kumar J., Tomar M., Umar A., Gupta V. (2014). Metal clusters activated SnO_2_ thin film for low level detection of NH_3_ gas. Sens. Actuators B.

[4] Goertz O., Popp A., Kolbenschlag J., Vogelpohl J., Daigeler A., Ring A., Lehnhardt M., Hirsch T. (2013). Intravital pathophysiological comparison of acid- and alkali-burn injuries in a murine model. J. Surg. Res..

[5] Kozawa S., Kakizaki E., Muraoka E., Koketsu H., Setoyama M., Yukawa N. (2009). An autopsy case of chemical burns by hydrochloric acid. Leg. Med..

[6] Amshel C.E., Fealk M.H., Phillips B.J., Caruso D.M. (2000). Anhydrous ammonia burns case report and review of the literature. Burns.

[7] Ly A., Luo Y., Cavaillès G., Olivier M.-G., Debliquy M., Lahem D. (2020). Ammonia sensor based on vapor phase polymerized polypyrrole. Chemosensors.

[8] Matsuguchi M., Fujii S. (2018). HCl gas sensor coating based on poly(*N*-isopropylacrylamide) nanoparticles prepared from water-methanol binary solvent. Sensors.

[9] (2017). Occupational Safety and Health Administration, Permissible Exposure Limits/OSHA Annotated Table Z-1. https://www.osha.gov/dsg/annotated-pels/tablez-1.html#ppm1.

[10] Qi J., Xu X., Liu X., Lau K.T. (2014). Fabrication of textile based conductometric polyaniline gas sensor. Sens. Actuators B.

[11] Yun Y.J., Hong W.G., Choi N.J., Kim B.H., Jun Y., Lee H.K. (2015). Ultrasensitive and highly selective graphene-based single yarn for use in wearable gas sensor. Sci. Rep..

[12] Li W., Chen R., Qi W., Cai L., Sun Y., Sun M., Li C., Yang X., Xiang L., Xie D. (2019). Reduced graphene oxide/mesoporous ZnO NSs hybrid fibers for flexible, stretchable, twisted, and wearable NO_2_ E-textile gas sensor. ACS Sens..

[13] Han J.-W., Kim B., Li J., Meyyappan M. (2013). A carbon nanotube based ammonia sensor on cotton textile. Appl. Phys. Lett..

[14] Subbiah D.K., Mani G.K., Babu K.J., Das A., Rayappan J.B. (2018). Nanostructured ZnO on cotton fabrics—A novel flexible gas sensor & UV filter. J. Clean. Prod..

[15] Yun Y.J., Hong W.G., Kim H.J., Jun Y., Lee H.K. (2017). E-textile gas sensors composed of molybdenum disulfide and reduced graphene oxide for high response and reliability. Sens. Actuators B.

[16] Van der Schueren L., De Clerck K. (2012). Coloration and application of pH-sensitive dyes on textile materials. Color. Technol..

[17] Boerman J.-K., van Harberden J.-K., Pannek C., Schmitt K., Tarantik K.R., Bauersfeld M.-L., Wöllenstein J. (2018). Improvement methods for colorimetric gas sensor for use in indoor livestock farming. Proceedings.

[18] Van der Schueren L., De Clerck K., Brancatelli G., Rosace G., Van Damme E., De Vos W. (2012). Novel cellulose and polyamide halochromic textile sensors based on the encapsulation of Methyl Red into a sol–gel matrix. Sens. Actuators B.

[19] Staneva D., Betcheva R., Chovelon J.-M. (2007). Optical sensor for aliphatic amines based on the simultaneous colorimetric and fluorescence responses of smart textile. J. Appl. Polym. Sci..

[20] Owyeung R.E., Panzer M.J., Sonkusale S.R. (2019). Colorimetric gas sensing washable threads for smart textiles. Sci. Rep..

[21] Schoolaert E., Hoogenboom R., De Clerck K. (2017). Colorimetric nanofibers as optical sensors. Adv. Funct. Mater..

[22] Van der Schueren L., Mollet T., Ceylan Ö., De Clerck K. (2010). The development of polyamide 6.6 nanofibres with a pH-sensitive function by electrospinning. Eur. Polym. J..

[23] Zhang C., Li Y., Wang W., Zhan N., Xiao N., Wang S., Li Y., Yang Q. (2011). A novel two-nozzle electrospinning process for preparing microfiber reinforced pH-sensitive nano-membrane with enhanced mechanical property. Eur. Polym. J..

[24] Agarwal A., Raheja A., Natarajan T.S., Chandra T.S. (2012). Development of universal pH sensing electrospun nanofibers. Sens. Actuators B.

[25] Kim S.-H., Bae J.-S. (2013). Halochromic chemosensor prepared by pyran-based nanofibers. Fibers Polym..

[26] Pakolpakçıl A.E., Karaca B.B. (2018). Investigation of a natural pH-indicator dye for nanofibrous wound dressings. Proceedings of the IOP Conference Series: Materials Science and Engineering, AUTEX.

[27] Geltmeyer J., Vancoillie G., Steyaert I., Breyne B., Cousins G., Lava K., Hoogenboom R., De Buysser K., De Clerck K. (2016). Dye modification of nanofibrous silicon oxide membranes for colorimetric HCl and NH_3_ sensing. Adv. Funct. Mater..

[28] Jeevarathinam A.S., Varathan E., Ravindran E., Somanathan N., Subramanian V., Mandal A.B., Sudha J.D., Ramakrishnan R. (2013). A solution processable fluorene–fluorenone oligomer with aggregation induced emission enhancement. Chem. Commun..

[29] Suleymanov A.A., Doll M., Ruggi A., Scopelliti R., Fadaei-Tirani F., Severin K. (2020). Synthesis of tetraarylethene luminogens by C− H vinylation of aromatic compounds with triazenes. Angew. Chem. Int. Ed..

[30] Oh B.M., Noh H.L., Gwon S.-Y., Park Y.K., Cho N., Lee W., Kim S.-H., Kim J.H. (2018). Some properties of a new D-π-A dye based on hydroxyl-methoxybenzene donor and isophorone acceptor moiety: Effects of anion, ethylamine and temperature. Dyes Pigment..

[31] Park Y.K., Oh B.M., Jo A.R., Han J.H., Lim J.Y., Oh H.J., Lim S.J., Kim J.H., Lee W.S. (2020). Fabrication of colorimetric textile sensor based on rhodamine dye for acidic gas detection. Polymers.

[32] Oh H.J., Yeang B.J., Park Y.K., Choi H.J., Kim J.H., Kang Y.S., Bae Y., Kim J.Y., Lim S.J., Lee W. (2020). Washable colorimetric nanofiber nonwoven for ammonia gas detection. Polymers.

[33] Nobbs J.H. (1985). Kubelka–Munk theory and the prediction of reflectance. Rev. Prog. Color. Relat. Top..

[34] Mokrzycki W.S., Tatol M. (2011). Colour difference ∆E—A survey. Mach. Graph. Vis..

[35] ISO (2006). Textiles—Tests for Colour Fastness—Part C10: Colour Fastness to Washing with Soap or Soap and Soda.

[36] KSA (2015). Test Method for Determination of Aryl Amine Level on the Dyestuff and Dyed Products.

[37] KSA (2014). Test Method for the Determination of Allergenous Dyes in Textiles.

[38] KSA (2009). Textiles—Determination of Formaldehyde—Part 1: Free and Hydrolized Formaldehyde (Water Extraction Method).

[39] KSA (2009). Textiles—Determination of pH of Aqueous Extract.

[40] Kessler M.A., Wolfbeis O.S. (1991). New highly fluorescent ketocyanine polarity probes. Spectrochim. Acta Part A.

[41] Soller B.R. (1994). Design of intravascular fiber optic blood gas sensors. IEEE Eng. Med. Biol. Mag..

[42] Van der Schueren L., Hemelsoet K., Van Speybroeck V., De Clerck K. (2012). The influence of a polyamide matrix on the halochromic behaviour of the pH-sensitive azo dye Nitrazine Yellow. Dyes Pigm..

