# Supplementary material for: Colorimetric Textile Sensor for the Simultaneous Detection of NH3 and HCl Gases
Source: Polymers (Basel). 2020 Nov 4;12(11):2595. doi: 10.3390/polym12112595 (PMC7694299; doi:10.3390/polym12112595)
Supplement: Supplementary file 1 [file polymers-12-02595-s001.pdf]

# Supplementary data

## Colorimetric Textile Sensor for the Simultaneous Detection of $NH_3$ and HCl Gases

Young Ki Park [1,†], Hyun Ju Oh [2,†], Jong Hyuk Bae [2], Jee Young Lim [2], Hee Dong Lee [2], Seok Il Hong [2], Hyun Sik Son [1], Jong H. Kim [3,*], Seung Ju Lim [4,*] and Woosung Lee [2,*]

[1]*Test-Bed Research Center, Korea Dyeing & Finishing Technology Institute (DYETEC), Daegu 41706, Korea;* parkyk@dyetec.or.kr (Y.K.P), hsson95@dyetec.or.kr (H.S.S)

[2]*Advanced Textile R&D Department, Korea Institute of Industrial Technology (KITECH), Ansan 15588, Korea;* hjoh33@kitech.re.kr (H.J.O), baejh@kitech.re.kr (J.H.B), specialg@kitech.re.kr (J.Y.L), lhd0121@kitech.re.kr (H.D.L), redstone@kitech.re.kr (S.I.H)

[3]*Department of Molecular Science and Technology, Ajou University, Suwon, 16499, Korea*

[4]*Department of Advanced Materials Engineering for Information & Electronics, Kyung Hee University, Yongin, 17104, Korea*

[†] *These authors contributed equally to this work*

[*]Correspondence author: jonghkim@ajou.ac.kr (J.H.K.), limsj5404@khu.ac.kr (S.J.L.), wslee@kitech.re.kr (W.S.L.)

**Table of contents**

**Table S1.** Effect of VOCs on the fabricated textile sensor based on mixed dyes.

**Table S2.** Content of hazardous materials of fabricated textile sensor.

**Table S1.** Effect of VOCs on the fabricated textile sensor based on mixed dyes

| | Before exposure | After exposure |
|---|---|---|
| **Formaldehyde** | 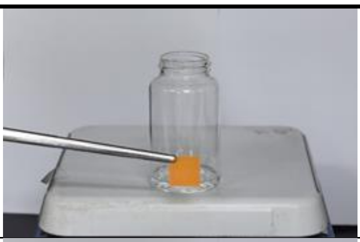 | 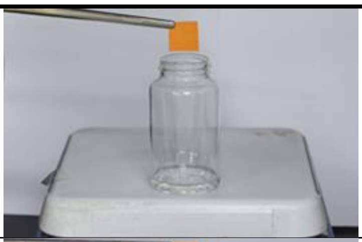 |
| **Ether** | 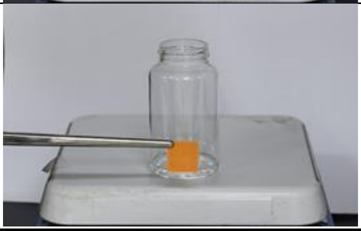 | 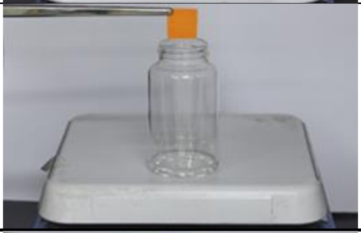 |
| **Methylene chloride** | 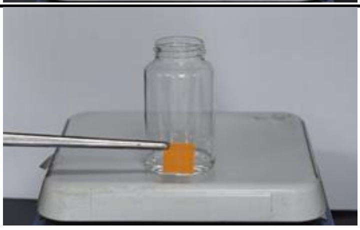 | 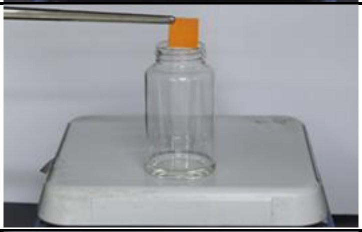 |
| **Acetone** | 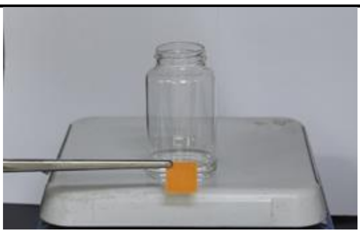 | 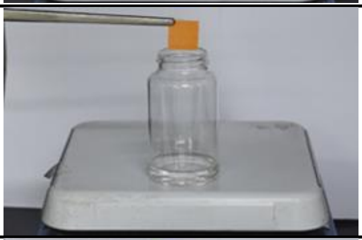 |
| **Methanol** | 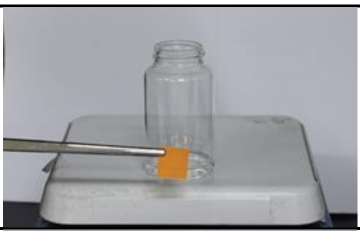 | 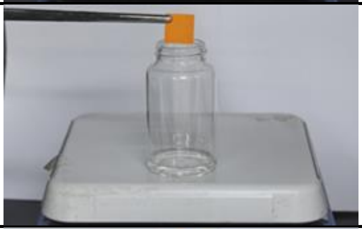 |
| **Ethanol** | 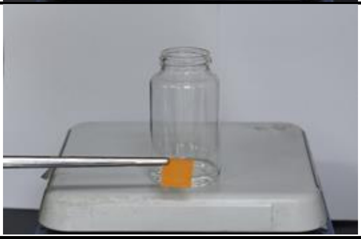 | 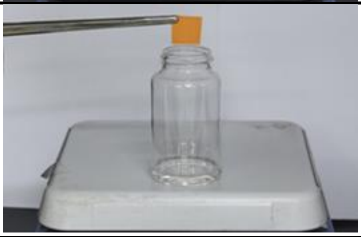 |
| **Toluene** | 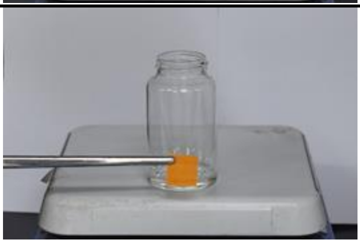 | 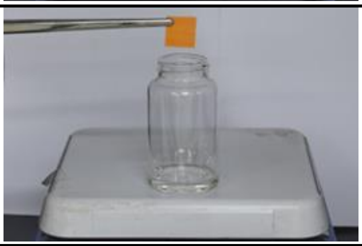 |

**Table S2.** Content of hazardous materials of fabricated textile sensor

| Test conducted | | Content (mg/kg) |
|---|---|---|
| Arylamines (Detection Limit 5 mg/kg) | 4-Aminobiphenyl | < 5 |
| | Benzidine | < 5 |
| | 4-Chloro-o-toluidine | < 5 |
| | 2-Naphthylamine | < 5 |
| | o-Aminoazotoluene | < 5 |
| | 2-Amino-4-nitrotoluene | < 5 |
| | 4-Chloroaniline | < 5 |
| | 2,4-Diaminoanisole | < 5 |
| | 4,4'-Diamino-diphenylmethane | < 5 |
| | 3,3'-Dichlorobenzidine | < 5 |
| | 3,3'-Dimethoxybenzidine | < 5 |
| | 3,3'-Dimethylbenzidine | < 5 |
| | 4,4'-Methylenedi-o-toluidine | < 5 |
| | p-Cresidine | < 5 |
| | 4,4'-Methylene-bis-(2-chloroaniline) | < 5 |
| | 4,4'-Oxydianiline | < 5 |
| | 4,4'-Thiodianiline | < 5 |
| | o-Toluidine | < 5 |
| | 2,4-Toluylenediamine | < 5 |
| | 2,4,5-Trimethylaniline | < 5 |
| | o-Anisidine | < 5 |
| | 4-Aminoazobenzene | < 5 |
| | 2,4-Xylidine | < 5 |
| | 2,6-Xylidine | < 5 |
| Allergenic Disperse Dyes (Detection Limit 20 mg/kg) | Disperse Blue 1 | < 20 |
| | Disperse Blue 3 | < 20 |
| | Disperse Blue 7 | < 20 |
| | Disperse Blue 26 | < 20 |
| | Disperse Blue 35 | < 20 |

| | | |
|---|---|---|
| | Disperse Blue 102 | < 20 |
| | Disperse Blue 124 | < 20 |
| | Disperse Orange 1 | < 20 |
| | Disperse Orange 3 | < 20 |
| | Disperse Orange 37/76/59 | < 20 |
| | Disperse Orange 149 | < 20 |
| | Disperse Yellow 1 | < 20 |
| | Disperse Yellow 3 | < 20 |
| | Disperse Yellow 9 | < 20 |
| | Disperse Yellow 23 | < 20 |
| | Disperse Yellow 39 | < 20 |
| | Disperse Yellow 49 | < 20 |
| | Disperse Red 1 | < 20 |
| | Disperse Red 11 | < 20 |
| | Disperse Red 17 | < 20 |
| | Disperse Brown 1 | < 20 |
| Formaldehyde content (Detection Limit 16 mg/kg) | | Not detected |
| pH | | 7.2 |
| Total lead(Pb) content (Detection Limit 10 mg/kg) | | < 10 |
| Total cadmium(Cd) content (Detection Limit 10 mg/kg) | | < 10 |
